# Osteopetrorickets due to Snx10 Deficiency in Mice Results from Both Failed Osteoclast Activity and Loss of Gastric Acid-Dependent Calcium Absorption

**DOI:** 10.1371/journal.pgen.1005057

**Published:** 2015-03-26

**Authors:** Liang Ye, Leslie R. Morse, Li Zhang, Hajime Sasaki, Jason C. Mills, Paul R. Odgren, Greg Sibbel, James R. L. Stanley, Gee Wong, Ariane Zamarioli, Ricardo A. Battaglino

**Affiliations:** 1 Department of Mineralized Tissue Biology, The Forsyth Institute, Cambridge, Massachusetts, United States of America; 2 Department of Oral Medicine, Infection, and Immunity, Harvard School of Dental Medicine, Boston, Massachusetts, United States of America; 3 Department of Physical Medicine and Rehabilitation, Harvard Medical School, Boston, Massachusetts, United States of America; 4 Spaulding Rehabilitation Hospital, Boston, Massachusetts; 5 Department of Immunology and Infectious Diseases, The Forsyth Institute, Cambridge, Massachusetts, United States of America; 6 Division of Gastroenterology, Department of Medicine, Washington University School of Medicine, St. Louis, Missouri, United States of America; 7 Department of Cell and Developmental Biology, University of Massachusetts Medical School, Worcester, Massachusetts, United States of America; 8 CBSET Inc, Lexington, Massachusetts, United States of America; 9 Department of Biomechanics, Medicine and Rehabilitation, Faculty of Medicine of Ribeirão Preto, University of São Paulo, São Paulo, Brazil; University of Cincinnati College of Medicine, UNITED STATES

## Abstract

Mutations in sorting nexin 10 (Snx10) have recently been found to account for roughly 4% of all human malignant osteopetrosis, some of them fatal. To study the disease pathogenesis, we investigated the expression of Snx10 and created mouse models in which Snx10 was knocked down globally or knocked out in osteoclasts. Endocytosis is severely defective in Snx10-deficent osteoclasts, as is extracellular acidification, ruffled border formation, and bone resorption. We also discovered that Snx10 is highly expressed in stomach epithelium, with mutations leading to high stomach pH and low calcium solubilization. Global Snx10-deficiency in mice results in a combined phenotype: osteopetrosis (due to osteoclast defect) and rickets (due to high stomach pH and low calcium availability, resulting in impaired bone mineralization). Osteopetrorickets, the paradoxical association of insufficient mineralization in the context of a positive total body calcium balance, is thought to occur due to the inability of the osteoclasts to maintain normal calcium–phosphorus homeostasis. However, osteoclast-specific Snx10 knockout had no effect on calcium balance, and therefore led to severe osteopetrosis *without* rickets. Moreover, supplementation with calcium gluconate rescued mice from the rachitic phenotype and dramatically extended life span in global Snx10-deficient mice, suggesting that this may be a life-saving component of the clinical approach to Snx10-dependent human osteopetrosis that has previously gone unrecognized. We conclude that tissue-specific effects of Snx10 mutation need to be considered in clinical approaches to this disease entity. Reliance solely on hematopoietic stem cell transplantation can leave hypocalcemia uncorrected with sometimes fatal consequences. These studies established an essential role for Snx10 in bone homeostasis and underscore the importance of gastric acidification in calcium uptake.

## Author Summary

We found that Snx10, a molecule expressed in osteoclasts, was also expressed in the stomach. Studies in tissue specific or global knock-down mice showed that Snx10 deficiency resulted in a phenotype that was a consequence of deficiencies in both osteoclasts and gastric zymogenic cells. Our studies add to a growing list of genes, including atp6i (*Tcirg1*), whose expression is required both in bone and stomach to maintain normal gastric acidification and calcium absorption. This work provides additional insight into the mechanisms governing the regulation of bone accrual by the gastrointestinal tract. Because osteopetrorickets has not been described clinically in Snx10-related osteopetrosis, these findings highlight the importance of considering impaired acidification in both stomach and bone in osteopetrotic patients with mutations in *SNX10* and other genes with similar patterns of expression and activities. Because defects in gastric differentiation and/or gastric acidification may cause or contribute to hypocalcemia, bone insufficiency, and early death, our results suggest that dietary calcium supplementation could be a life-saving intervention in these patients.

## Introduction

The function of the bone-resorbing osteoclast is highly dependent on vesicular trafficking pathways [1]. Endocytosis and intracellular trafficking of the endocytosed material are required for many osteoclast functions, including creation of the ruffled border; secretion of ions and proteases to digest bone; to engulf the digested material; to move it across the cell by transcytosis; and to secrete the products of digestion [2,3]. Disruption (genetic or pharmacological) of osteoclastic vesicle transport abolishes resorptive activity [1]. For example, osteoclasts from human patients and from rats deficient in Plekhm1, a protein with a critical function in vesicular transport in osteoclasts, develop osteopetrosis and have osteoclasts with secretory defects and which lack ruffled borders [4].

Members of the sorting nexin (Snx) family of proteins are known to mediate endosomal sorting, endocytosis, recycling of membrane proteins, and trafficking between various endosomes and Golgi apparatus [5]. The Snx family consists of a diverse group of cytoplasmic and membrane-associated proteins that are unified by a common phospholipid-binding motif, the PX domain. They participate in protein sorting and membrane trafficking by means of protein-protein complexes and protein-lipid interactions. [5]. Overexpression of one Snx family member, Snx10, induced giant vacuoles in mammalian cells [6].

During investigations of genes differentially expressed during RANKL-induced osteoclast differentiation, we found *Snx10* to be very strongly upregulated both *in vitro* and *in vivo* [7]. Immunohistochemical analysis of mouse embryo sections showed expression in long bone, calvariae, and developing teeth. Snx10 was expressed in cells that also express tartrate-resistant acid phosphatase (TRAP), demonstrating osteoclastic localization [7]. *Snx10* silencing inhibited formation of resorption pits on hydroxyapatite and also TRAP secretion [7]. Taken together, these results indicate that *Snx10* is expressed in osteoclasts and is required for osteoclast activity *in vitro*.

In 2012, *SNX10* mutations were discovered in patients with infantile autosomal recessive osteopetrosis. One was a point mutation that caused a single amino acid change in a highly conserved residue, R51Q, [8] and one introduced a premature stop codon [9]. Osteoclasts from these patients showed reduced resorptive capacity and altered endosomal pathways [8]. In 2013, nine novel mutations in *SNX10* were then described in 14 autosomal recessive osteopetrosis (ARO) patients, and together, *SNX10* mutations are now known to accounting for about 4% of known ARO cases, roughly the same proportion as mutations in the RANK-RANKL pathway or in *OSTM1* [10]. Most patients with *SNX10* mutations benefited from hematopoietic stem cell transplants (HSCT). However, some patients exhibited symptoms consistent with osteopetrorickets and did not experience improvement after HSCT, which suggests that bone may not be the only site of expression [10].

Several molecules in the acid producing system are expressed in both bone and stomach cell types, including Atp6i, Clc-7 and Ae2. ATP6i [11] and Ae2 [12–14] loss-of-function mutants develop osteopetrorickets due to simultaneous acidification defects leading to osteoclast dysfunction and impaired calcium absorption. Osteopetrorickets has been described in both humans and in mice and it is often fatal in infants. It manifests as the seemingly paradoxical combination of dense, sclerotic bones, but with defective mineralization of hypertrophic cartilage and of osteoid, often in the presence of elevated parathyroid hormone, alkaline phosphatase, and decreased 1,25-dihydroxyvitamin D levels [15,16]. While rickets was once considered a rare complication of osteopetrosis, it has been suggested that osteopetrorickets is more common than previously thought [17–19].

Given its critical role in osteoclast function [7], it seemed likely that Snx10 may also regulate acid production in the stomach. To study the role of Snx10 in bone homeostasis *in vivo* we generated strains of mice that carry either a global knockdown of Snx10 or an osteoclast-specific knockout. We report here that *Snx10* is highly expressed, not only in osteoclasts, but also in gastric epithelium, leading to osteoclastic and gastric acidification defects, similar to the Tcirg1-deficient oc/oc mice [11]. We also show that osteoclast-specific knockout of *Snx10* led to severe osteopetrosis, but without rickets, and that dietary calcium supplementation rescued mice with a global knockdown of *Snx10* from the rachitic phenotype and prevented juvenile lethality, indicating that this simple remedy should always be considered clinically.

## Results

### Consequences of global Snx10 deficiency *in vivo*: osteopetrosis and rickets

In order to study the role of Snx10 in osteoclast function and bone homeostasis *in vivo* we generated mice from KOMP gene-trapped ES cells (PG00216_Z_2_C06, S1A Fig.). This targeting construct places a selection cassette and several recombination sites within intron 3, flanks exons 4 and 5 with loxP sites, and disrupts transcription. PCR genotyping (S1B Fig.) and mRNA levels (S1C Fig.) validated correct insertion and revealed an 86% reduction in Snx10 levels in bones from the resulting, homozygous mice compared to wildtype littermates (relative expression = 0.14 vs. 1.03, n = 4 per group, p<0.05, S1C Fig.). We have designated these mice *Snx10* ^*Neo-f/Neo-f*^ (see EXPERIMENTAL PROCEDURES) and we hereafter refer to them *as* “Snx10 KD” for Snx10 knockdown to reflect the global insufficiency of *Snx10* expression.

**Fig 1:**
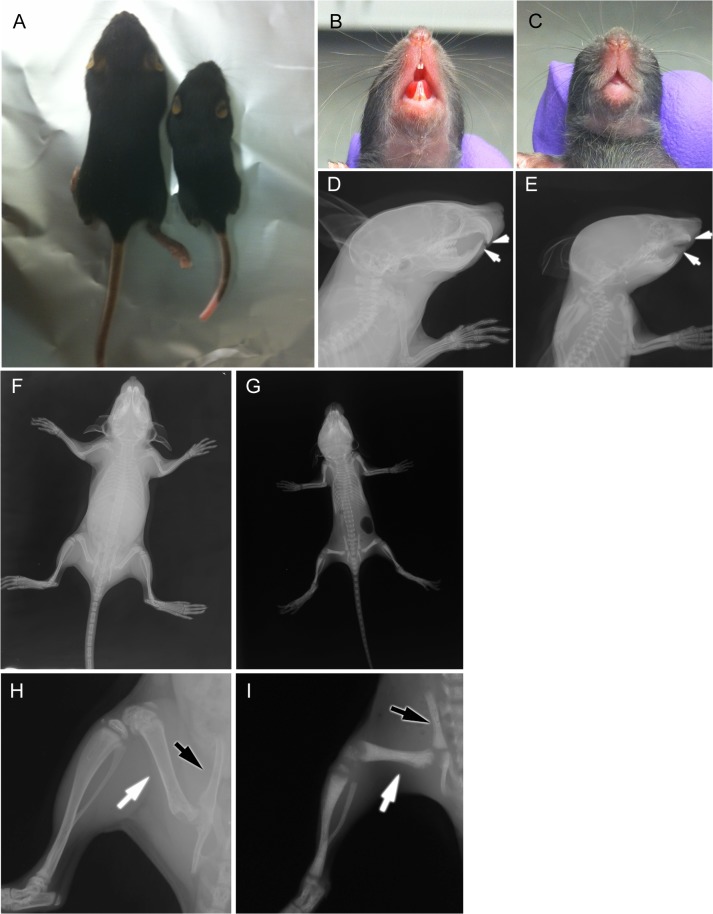
Morphological and radiographic examination of Snx10 KD mice. A) 2-week-old Snx10 KD mice are growth retarded. 2-week-old Snx10 KD mice (C and E) have a tooth eruption defect compared to their WT littermates (B and D). The white arrows (D and E) demonstrate the presence of tooth buds that failed to erupt in the KD mice. Radiographic images demonstrate that 3-week-old Snx10 KD mice (G and I) have bones that lack marrow cavities. Compare the femur (white arrow) and pelvis (black arrow) with WT (F and H) mice. The metaphyseal cupping in the Snx10 KD femur and tibia (I) are consistent with rickets.

**Fig 2:**
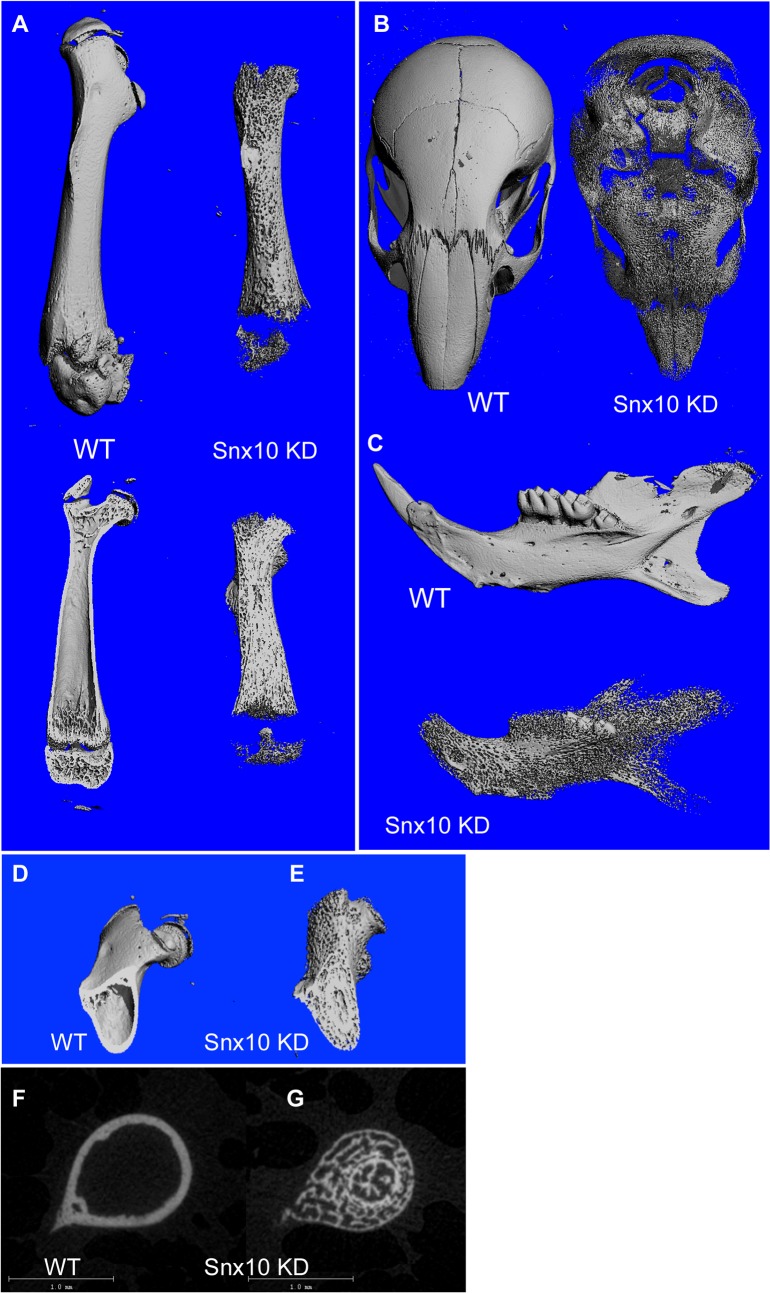
Micro CT analysis of Snx10 KD mice. (A, top) Surface images of the femur demonstrate a deficiency of mineralized cortical bone. Longitudinal mid-plane images of the femur (A, bottom) show a marrow cavity filled with unresorbed bone in Snx10 KD mice. Other examined bones: head, (B) and mandible (C) also were deficient in cortical bone, producing a “moth-eaten” appearance. Cross sectional micro CT images of femur (D, E, F and G) reveal the presence of an inner ring of cortex-like (i.e., denser) bone in Snx10 KD bones (E and G).

Snx10 KD mice die between 3 and 4 weeks post-partum. By 14 days of age Snx10 KD mice exhibited severe growth retardation compared to WT or heterozygous controls (Fig. 1A), with failed tooth eruption (compare Fig. 1B and 1C). The overall skeletal development was impaired, with higher radio-density in the 3-week-old Snx10 KD mice (Fig. 1E, 1G and 1I) compared with the wild-type mice (Fig. 1D, 1F and 1H). Further skeletal examination of 3 week-old mice by micro-CT (Fig. 2) confirmed that the long bones of Snx10 KD mice were filled with unresorbed trabecular bone and lacked marrow spaces, consistent with a severely osteopetrotic phenotype. Micro-CT analysis of the femur, skull, and mandible performed in Snx10 KD mice (n = 4) and WT littermates (n = 3) confirmed the osteopetrotic phenotype. In fact, Snx10 KD mice had significantly higher BV/TV (0.31 +/− 0.052 vs 0.070 +/− 0.004, P = 0.0006), higher trabecular number (15.17 +/− 2.92 1/mm vs 2.29 +/− 0.004 1/mm, P = 0.0007) and significantly reduced trabecular spacing (0.061 +/− 0.009 mm vs 0.391 +/− 0.042mm, P = 1.97535E-05) than WT. Trabecular thickness values, on the other hand, were not different (0.032 +/− 0.002 mm for the Snx10 KD, 0.031 +/− 0.004 mm for the WT, p = 0.793). The abundance of trabecular bone suggests that the observed phenotype is mainly the result of an osteoclast defect. In addition, all examined Snx10 KD bones exhibited a thinned or absent cortex, producing a dramatic “moth-eaten” appearance (Fig. 2A, 2B, and 2C). Finally, analysis both by radiograph and by micro-CT of transverse sections of long bones (femur, tibia, humerus) revealed an inner ring of cortex-like (denser) bone within the trabecular bone (shown in the femur, Fig. 2E and 2G). This is reminiscent of the classic “bone-in-bone” or "bone within bone" appearance, a typical radiologic finding in osteopetrosis, usually reserved for the vertebral column [15,17,20–22]. Radiographs also revealed metaphyseal fraying and cupping (Fig. 1I), indicative of rickets superimposed on osteopetrosis, or “osteopetrorickets.” To confirm this, we compared Snx10 KD mice with *Tcirg1* (Atp6i) KO mice [23], which display both hypocalcemia and osteopetrorickets [11]. Radiograph and micro-CT analysis (S2 Fig.) demonstrated that that the *Tcirg1* KO mice were strikingly similar to the Snx10 KD mice by radiograph and micro-CT, with lack of cortical bone, metaphyseal cupping and fraying, and non-mineralized condyles and articulations, all characteristic of rickets. Moreover, biochemical investigations showed hypocalcaemia and high PTH together with low levels of serum 25-hydroxy vitamin D (see below). Based on our own findings and reports on mice and humans with *TCIRG1* mutations, we conclude that the Snx10 KD mice have a phenotype of osteopetrosis with super-imposed rickets: osteopetrorickets [17,24].

Analysis of bone mineral density by DXA demonstrated that BMD is reduced ∼14% in Snx10 KD mice compared to WT mice (0.0337 +/− 0.002 g/cm^2^ vs. 0.0393 +/− 0.003 g/cm^2^, respectively. n = 6 per group, P = 0.02). Furthermore, BMC is reduced 43% (0.114 +/− 0.043 g vs. 0.199 +/− 0.034 g, Snx10 KD and WT respectively, P = 0.006). The greater reduction in BMC is likely a reflection of both reduced BMD and the small size of the Snx10 KD mice

**Fig 3:**
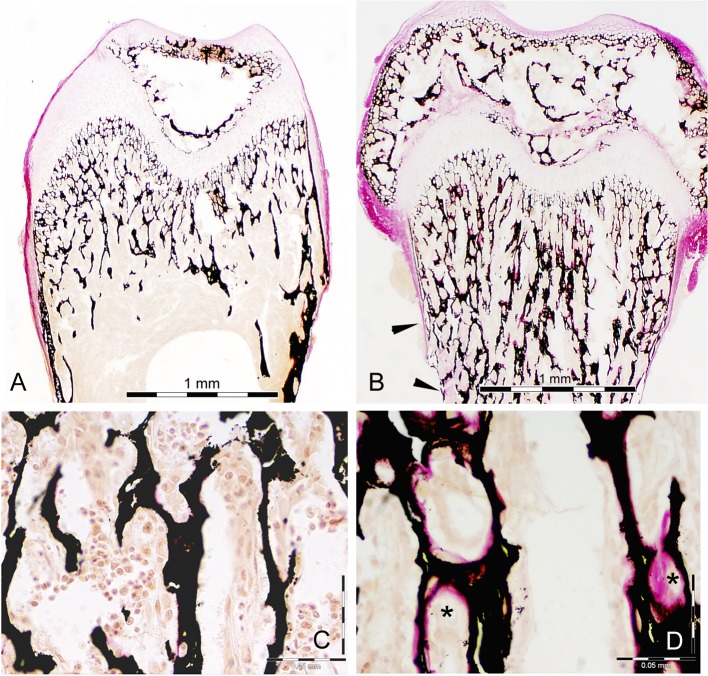
Histology of bone from WT and Snx10 KD mice. Low magnification of Von Kossa/van Gieson staining (A and B) of femur longitudinal sections from 3 week-old mice demonstrate endosteum filled with unresorbed cartilage (purple stained trabecular cores) and thin cortical bone (black arrowheads) in Snx10 KD mice (B). Higher magnification (C and D) reveals non-mineralized osteoid (pink-stained surface, indicated with asterisks), covering the mineralized trabeculae (black) from Snx10 KD bones (D) Bar = 0.05 mm

These observations were further confirmed by histological analysis of femora from 3-week-old mice. Low magnification images (Fig. 3A and 3B), demonstrated: 1) a marrow cavity almost completely filled with unresorbed, poorly mineralized cartilage, and 2) thinning of cortical bone (black arrowheads) in the Snx10 KD mice. High magnification images of von Kossa/van Gieson stained undecalcified longitudinal sections of femora from WT and Snx10 KD mice confirmed the presence of numerous trabeculae within the bone marrow space of Snx10 KD bones (Fig. 3D). Interestingly, the van Gieson counterstain also revealed the presence of non-mineralized osteoid on the surface of the trabeculae (Fig. 3D). Histomorphometry performed on longitudinal femoral sections confirmed a significant increase in growth plate thickness (GpTh,) in Snx10 KD mice compared to WT mice (0.286 +/− 0.062 mm and 0.175 +/− 0.010 mm, respectively, P = 0.04, n = 3 per group, S2 Table). We also detected a significant increase in Osteoid volume per Bone volume (OV/BV, %) in Snx10 KD mice (26.97 +/− 8.19% and 2.88 +/− 1.63%, respectively, P = 0.01) and in Bone volume / Tissue volume (BV/TV, %) (20.69 +/− 2.15% for the WT and 28.11 +/− 0.98% for the Snx10 KD, P = 0.02, S2 Table). Histomorphometry performed on sections from lumbar vertebral bodies (S4 Table) and from skull base/floor bones (S5 Table) show similar results. Put together, these results confirm a combined phenotype in Snx10 KD mice characterized by both a bone mineralization defect (rickets) and osteopetrosis due to defective osteoclast resorption.

We performed mechanical testing of tibial diaphyses (three-point-bending) to obtain the following parameters: maximal load, stiffness, energy to failure, and maximal displacement, which together provide an accurate picture of the biomechanical status of bone [25]. We found that the tibiae of Snx10 KD mice have significantly lower maximal load (P = 0.0025), stiffness (P = 0.001), energy to failure (P = 0.027), and greater maximal displacement (P = 0.05) compared to WT (n = 6, S1 Table). Therefore, compared to WT, the long bones of Snx10 KD mice are weaker, less mineralized, and easier to deform in response to mechanical stress. It should be noted that all the mentioned values (maximal load, stiffness, energy to failure and displacement) are dependent on bone size. Therefore the values obtained for bones from Snx10 global deficient mice (Snx10 KD) reflect the fact that they have different material properties and that they are smaller. These results are consistent with rickets, which leads to softening and weakening of the bones (osteomalacia) [24], whereas osteopetrosis alone results in hard, brittle bones due to the persistence of woven bone [22,26].

Taken together, these results show that Snx10 is essential for bone homeostasis and to maintain normal biomechanical properties of long bones.

### Snx10 deficiency inhibits osteoclast activity

An osteopetrotic phenotype can be the result of absent osteoclasts (*e*.*g*., RANKL knockout), which indicates a defect in osteoclast differentiation. Osteopetrosis can also result from impaired osteoclast function (*e*.*g*., c-Src knockout [27]). Histological analysis showed that bones from Snx10 KD mice have TRAP-positive osteoclasts (S2A Fig.), which suggests that Snx10 deficiency affects osteoclast function.

Due to the lack of marrow in Snx10 KD mice, we used splenocytes as a source of osteoclast precursors from WT and Snx10 KD mice for *ex vivo* differentiation experiments. Snx10 KD splenocytes gave rise to multinucleated osteoclasts, confirming that Snx10 deficiency does not inhibit osteoclast formation (S2C Fig.). In pit formation assays we found that, although the Snx10-deficient cells gave rise to TRAP-positive multinucleated osteoclasts, the total area resorbed by Snx10 KD osteoclasts was reduced by 94% (S2D Fig., top panel) (3.99 +/− 1.41 mm^2^ for the WT, 0.26 +/−0.15 mm^2^ for the *Snx10 KD*, n = 6, P = 0.04), in agreement with our knockdown results [7]. Finally, infection of Snx10 KD splenocytes with a retrovirus expressing Snx10 reintroduced Snx10 expression (S2D Fig., bottom panel) and corrected the pit formation defect (2.25 +/− 0.62 mm^2^ n = 6, p = 0.008), confirming that Snx10 deficiency is responsible for the resorption defect (S2D Fig., top and middle panels).

To determine the effect of Snx10 deficiency on bone turnover, we assessed levels of serum markers for bone formation (osteocalcin) and resorption (collagen I C-telopeptide; CTX). We found no significant differences in osteocalcin (18.35 +/− 1.81 ng/ml in the WT and 15.09 +/− 0.18 ng/ml in the Snx10 KD, n = 6, P = 0.127), suggesting that the bone phenotype observed in Snx10 KD is not primarily a bone formation defect. In contrast, serum CTX levels were significantly elevated in *Snx10 KD* mice compared to WT mice (88.56 +/− 5.67 ng/ml in the WT and 220.09 +/− 16.77 ng/ml in *Snx10 KD*, n = 6, P = 0.0002). This seemingly contradictory finding is consistent with reports in other osteopetrosis models [28–30], and may be due to the very large bone area resulting in a much larger total number of osteoclasts in the Snx10 KD mice (see Discussion).

### Impaired gastric acidification with subsequent hypocalcemia leads to osteopetrorickets in Snx10 KD mice

**Fig 4:**
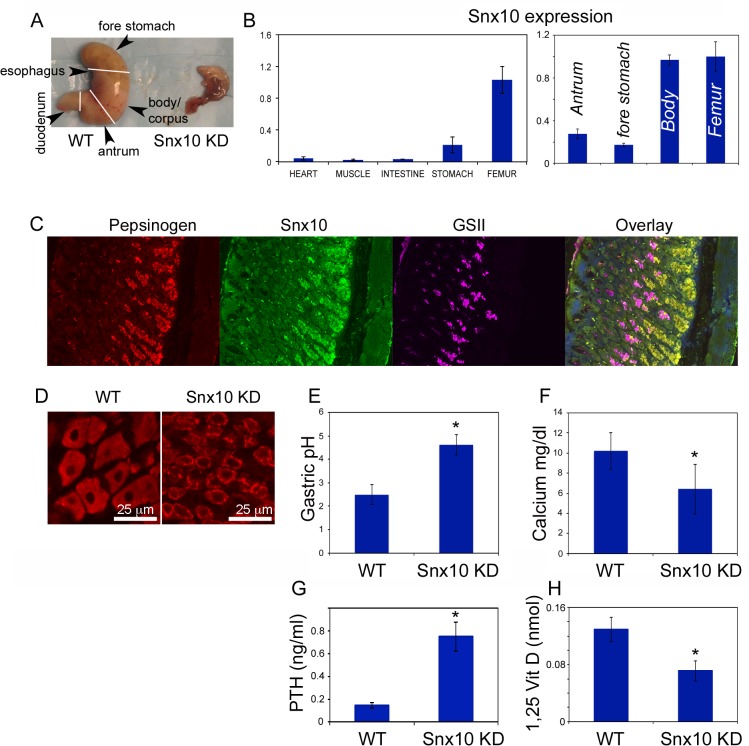
Snx10 is expressed in the stomach and is required for gastric acid production and calcium homeostasis. A) Representative stomachs from 3-week old Snx10 KD mice (n = 6) and WT littermates (n = 6). Snx10 KD mice have abnormal stomachs. B) mRNA expression of Snx10 (left panel) is mainly observed in stomach and bone. Within the stomach (right panel) expression of Snx10 mRNA is mainly observed in the body/corpus of the stomach ("Body"), where levels are comparable to the femur; expression is lower in the antrum and the non-glandular forestomach. C) Immunofluorescence images from stomach sections stained for pepsinogen (a zymogenic cell marker), Snx10 and GSII (a mucous neck cell marker) shows Snx10-specific staining (green) in zymogenic cells. D) Sections of the body of the stomach stained for the parietal cell marker VEGF-B (red), show that Snx10-deficient parietal cells are smaller, have abnormal nuclear morphology, increased nuclear to cytoplasmic ratio, and an abnormal cellular distribution of VEGF-B. E) Gastric pH is significantly elevated in Snx10 KD mice indicating that Snx10 is required for gastric acid production (* P<0.05). F) Serum calcium is reduced in Snx10 KD mice, indicating that Snx10 is required for calcium homeostasis (*P<0.05). G) Serum parathyroid hormone (PTH) is significantly elevated in Snx10 KD (* P = 0.001). H) 1,25-dihydroxyvitamin D levels are reduced in Snx10 KD mice (* P = 0.01).

Morphological examination of stomachs (n = 6) showed that Snx10 KD mice have an abnormal digestive tract, with stomachs prone to hemorrhagic necrosis (Fig. 4A), suggesting that Snx10 deficiency may result in a functionally impaired digestive system. Since Snx10 is required for extracellular acidification in osteoclasts, we hypothesized that it might also be required for gastric acid production and subsequent normal intestinal calcium absorption. To test this, we first examined Snx10 expression by qPCR analysis and found it to be expressed in the stomach and bone, but not in heart, muscle or intestine (Fig. 4B, *left panel*). In the stomach, expression was restricted to the oxyntic mucosa of the body/corpus (Fig. 4B, *right panel*), the region producing gastric acid. Expression of Snx10 mRNA in the stomach of Snx10 KD mice is reduced by 78% compared to WT. This is a similar reduction to that in bone (S1C Fig.). Immunofluorescence of stomach sections confirmed that Snx10 is normally present predominately in the intracellular secretory granules of zymogenic chief (ZC) cells (Fig. 4C, WT). Immunofluorescence shows that Snx10 antibody labels ZC cells at the base of the gastric units only in WT mice (S4A Fig., *bottom)*. ZC in WT are large cells containing apical cytoplasm filled with secretory granules which show intense labeling for the marker, GIS, whereas in Snx10 KD mice, ZCs are smaller and have sparser, more punctate granules with weak GIS staining (S4A Fig.). This resembles other mouse models with defects in secretory granule maintenance/formation, such as the *Mist1^-/-^* mouse [31,32]. GSII labels mucous neck cells, which are interspersed between parietal cells and define the mid/neck region of each gastric unit. Their abundance and location in Snx10 KD mice are not distinguishable from WT. H&E staining (S4B Fig.) suggests a small size and high nuclear:cytoplasmic ratio of parietal cells in Snx10 KD mice. The ZCs, lacking their abundant apical granules, are also smaller, and there are increased parietal cells in the base (ZC) zone (S4B Fig., bottom, black arrowheads). Stomach sections were also stained for the parietal cell marker VEGF-B [33]. We confirmed that, compared to their WT counterparts, Snx10-deficient parietal cells are smaller and have an increased nuclear to cytoplasmic ratio (Fig. 4D). Also, VEGF-B is focused in a ring-like cytoplasmic pattern in Snx10-deficient parietal cells, whereas it is broadly distributed baso-laterally in WT cells, suggesting a defect in cellular protein trafficking.

To study possible functional consequences of Snx10 deficiency in the stomach, we measured gastric pH in WT and Snx10 KD stomachs. We detected a significant increase in stomach pH of Snx10 KD mice compared to WT mice (Fig. 4E, 4.62 +/− 0.43 vs. 2.50 +/−0.32, respectively, n = 5 per group, P<0.001). Taken together, these findings demonstrate that Snx10 is expressed in the stomach and its expression is required for stomach acidification. Because impaired stomach acidification reduces calcium absorption by the intestine, we investigated whether Snx10 was required for maintaining calcium homeostasis, which could explain the rachitic aspects of the phenotype.

Assessment of serum calcium confirmed that Snx10 KD mice are severely hypocalcemic compared to their WT littermates (KO 6.40 +/− 2.46 mg/dl vs WT 10.21 +/− 1.81 mg/dl, n = 6 per group, P = 0.01, Fig. 4F). They also had higher serum parathyroid hormone PTH (0.748 +/− 0.127 ng/ml, Fig. 4G) compared to the WT (0.142 +/− 0.023 ng/m, n = 3 per group, P = 0.001) and lower 1,25-dihydroxyvitamin D levels (0.071 +/− 0.014 nmol, Fig. 4H), compared to WT (0.129 +/− 0.017 nmol, n = 3 per group, P = 0.01); both typical manifestations of osteopetrorickets [17–19]. Taken together, these results suggest that the rachitic features of Snx10 KD are due to hypocalcemia caused by the acidification defect in the stomach.

**Fig 5:**
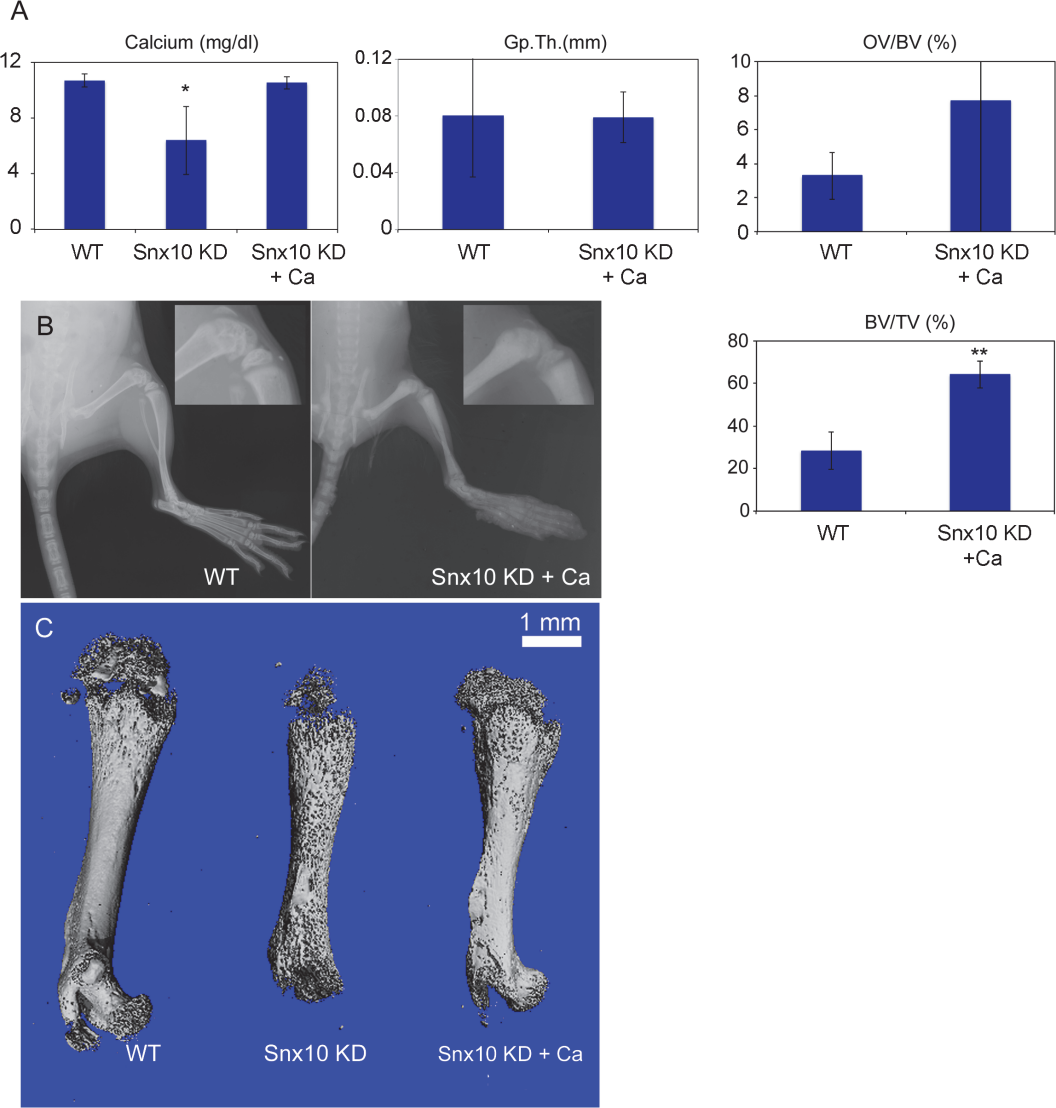
Calcium supplementation can normalize calcium homeostasis and correct the rickets phenotype in Snx10 KD mice. Serum calcium (A, left), GpTh (A, center) and OV/BV, (%;A, right) are normalized by calcium supplementation of Snx10 KD mice (Snx10 KD + Ca) (*, P<0.05, **, P<0.01). BV/TV(%; A, bottom right) is not normalized by calcium supplementation. B) Radiographic images of femora and tibiae show mineralization of femoral condyles (see inset) (C) Micro CT analysis of femora confirms partial re-mineralization of condyles and prevention of the “moth-eaten” cortical bone phenotype, as a result of calcium supplementation in Snx10 KD mice.

To confirm this, we performed calcium supplementation to rescue mice from the Snx10 KD rachitic phenotype. The diet included 2% calcium gluconate and 2,000 IU kg^-1^ vitamin D as described [11]. Due to the tissue damage seen in the stomachs of Snx10 KD mice we sought to improve calcium uptake by also giving sub-cutaneous injections of 2% calcium gluconate diluted in saline (50μl per day). Calcium supplementation was initiated at 14 days after birth and continued for 10 days. In a parallel survival experiment, mice (n = 5) were treated at 14 days after birth for 16 weeks. Calcium supplementation of Snx10 KD mice prevented the premature death seen at 3–4 weeks in the untreated mice. All mice receiving supplementation survived for the duration of the survival experiment. Treatment restored normocalcemia (10.52 +/− 0.43 mg/dl for treatment group vs. 10.70 +/− 0.46 mg/dl for the WT group, n = 6, P = 0.661) (Fig. 5A, *left*), bone mineral density (n = 6, P = 0.006) and bone mineral content (n = 6, P = 0.001). Radiographic analysis of femora demonstrated mineralization of the condyles and patellae in the calcium supplementation group (Fig. 5B, Snx10 KD+Ca *panel*) in contrast to the untreated Snx10 KD mice (Fig. 1I). Also, mutant mice receiving calcium supplementation lacked frayed or cupped metaphyseal plates (compare 1I and 5B), consistent with rescue from rickets. These results were further confirmed by histomorphometry. In fact, calcium supplementation of Snx10 KD mice restored growth plate thickness (GpTh,) and osteoid volume per bone volume (OV/BV, %) to values undistinguishable form WT (Fig. 5A, center and top right and S6 Table). Bone volume / tissue volume (BV/TV, %), on the other hand, remained significantly higher in the treated group compared to the WT (64.21 +/− 6.31% and 28.35 +/− 8.68%, respectively, P = 0.04, n = 3 per group, Fig. 5A, bottom right and S6 Table). We next analyzed femora by micro-CT (Fig. 5C) and confirmed restoration of cortical bone in the calcium supplementation group. Taken together, these results demonstrate that both the mortality and the mineralization defect observed in Snx10 KD mice were caused by hypocalcaemia and were prevented by calcium supplementation.

### Osteoclast-specific Snx10 deficiency *in vivo* results in osteopetrosis with no rickets

To determine the role of osteoclastic expression of Snx10 on bone homeostasis *in vivo*, we used the recombination sites in the gene trap (S1A Fig.). Snx10 KD mice were crossed with flippase-expressing mice to delete the selection cassette in exon 3, leaving a floxed allele that deleted exons 4 and 5 of Snx10 specifically in osteoclasts when crossed with mice expressing Cre under the control of the cathepsin K promoter (generously provided by Dr. Shigeaki Kato, Institute of Molecular and Cellular Biosciences, The University of Tokyo). Exons 4 and 5 encode the PX domain, which is essential for phospholipid binding. We designated this allele *Snx10* ^*OC-*^. *Snx10* ^*OC-/OC-*^ homozygous animals are hereafter referred to as “Snx10 OC KO”, for osteoclast knockout.

**Fig 6:**
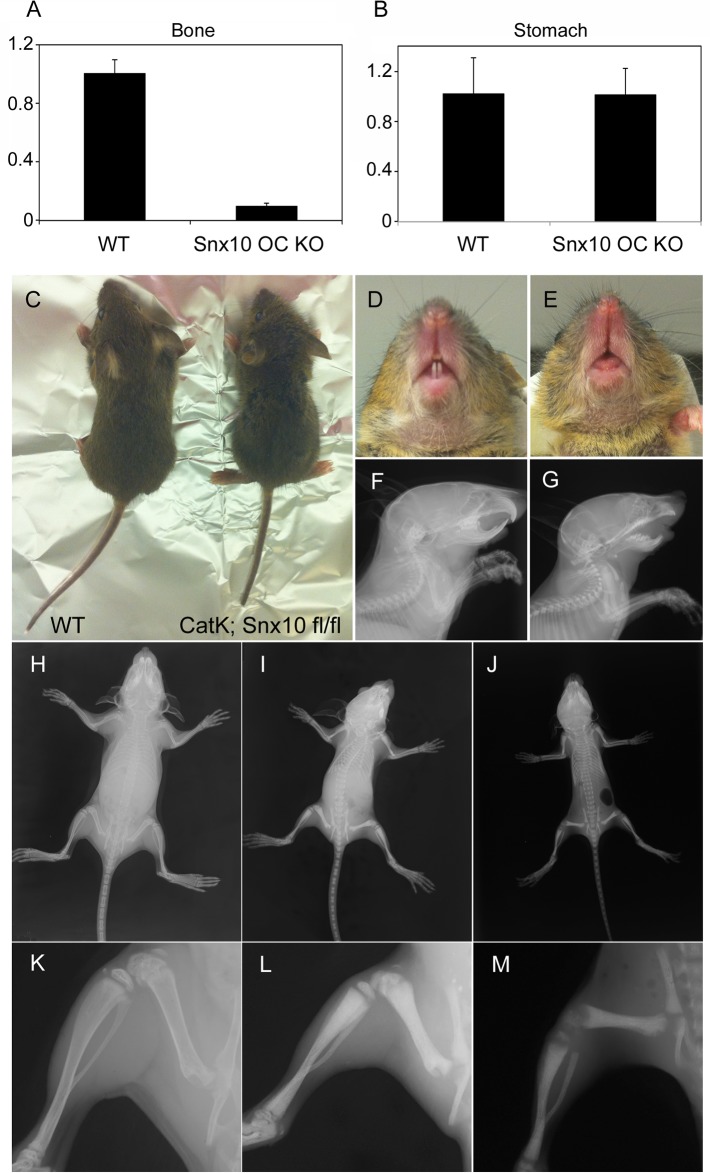
Snx10 OC KO mice are osteopetrotic: Expression, morphological and radiographic analysis. A) qPCR analysis of RNA from femur shows a 95% reduction in Snx10 expression in bones from Snx10 OC KO mice. B) Snx10 expression in the stomach is not affected. C) 3-week-old Snx10 OC KO mice (right) are growth retarded. E and G) 3-week-old Snx10 OC KO mice have a tooth eruption defect compared to their WT littermates (D and F). Radiographs (I and L) show that 3-week-old Snx10 OC KO mice have bones without marrow spaces and with a higher radio-density than WT counterparts (H and K) or Snx10 KD (J and M). The metaphyseal fraying and cupping seen in the Snx10 KD femur and tibia are not observed in the Snx10 OC KO mice (I and L), consistent with osteopetrosis with no rickets.

**Fig 7:**
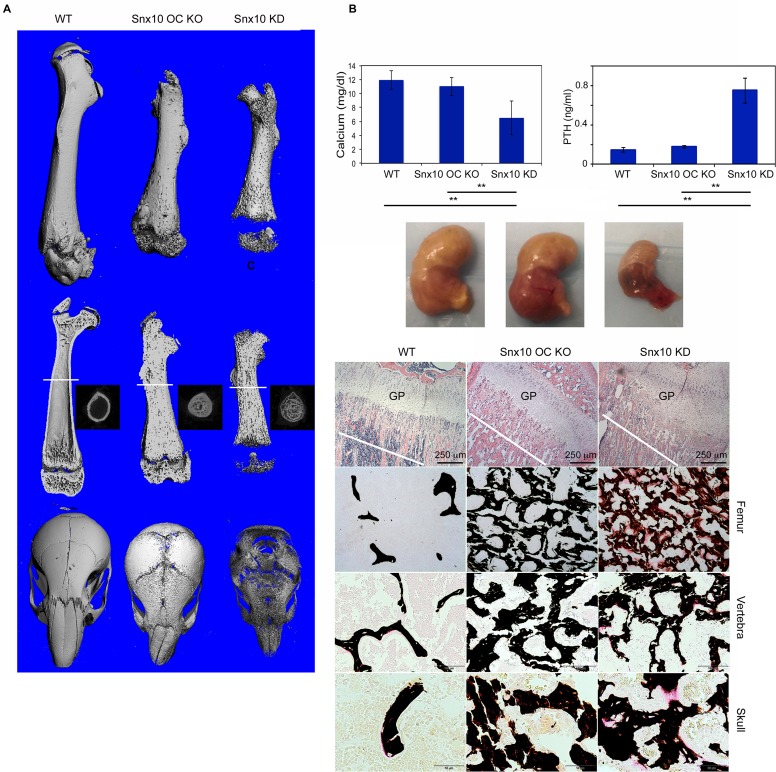
Histology and Micro CT analysis of Snx10 OC KO mice. A) Surface images of the femur (top), longitudinal mid-plane sections (middle) and skull images (bottom) show that osteoclast-specific Snx10 deficiency leads to severely osteopetrotic bone, but unlike the global deficiency it does not result in a lack of cortical bone. The white line in the longitudinal mid-plane sections (A, middle) indicates the location of the transverse micro-CT images were taken. The transverse images show that, in contrast to the Snx10 KD mouse, Snx10 OC KO mice have cortical bone. B) Snx10 OC KO mice are normo-calcemic, suggesting that that osteoclast-specific Snx10 deficiency does not have a major impact in calcium homeostasis. C) (top panel) H&E staining of longitudinal sections of the proximal femur show abundant unresorbed cartilage in Snx10 OC KO and Snx10 KD mice, typical of osteopetrosis. The growth plate (GP) is thicker in Snx10 KD mice. C) (FEMUR) Von Kossa/van Gieson staining of femur transverse sections close to the growth plate (cut at the depth of the white line shown in the upper panels) show numerous trabeculae in both Snx10 OC KO and Snx10 KD tibiae, confirming the osteopetrotic phenotype. However, while the trabeculae of Snx10 KD mice are not fully mineralized as evidenced by the pink-stained osteoid (right panels), the trabeculae of osteoclast-specific Snx10 KO mice are fully mineralized (bottom panels). Similar results were observed for vertebra (C. VERTEBRA panel) and *bones* of the skull base/floor (C. SKULL panel)

Snx10 OC KO mice showed a 95% reduction in Snx10 expression in bone (Fig. 6A); however expression in the stomach was not affected (Fig. 6B). Snx10 OC KO mice were viable and had survival times that did not differ from their WT littermates. However, by 3 weeks of age homozygotes exhibited mild growth retardation (Fig. 6C) with failure of tooth eruption (Fig. 6E and 6G versus 6D and 6F). Radiograph analysis showed higher radio-density but no metaphyseal plate widening, fraying or cupping of the tibia and femur, compared with the WT (Fig. 6H-M). DXA analysis of 9 week-old bones revealed a significant 30% increase in Snx10 OC KO mice compared to WT (n = 8, 0.074 g/cm^2^ vs. 0.057 g/cm^2^, P = 0.0001). Micro-CT analysis demonstrated the presence of cortical bone in the femur, and the skull surface was also mineralized. Consequently, there was no "moth-eaten" appearance (Fig. 7A, *top and bottom*). Similar to the Snx10 KD, the long bones of the Snx10 OC KO mice have marrow cavities filled with unresorbed bone (Fig. 7A, *center* and 7C). Therefore, osteoclast-specific Snx10 deficiency resulted in osteopetrosis.

Gastric pH was normal (3.06 +/− 0.3) and so was serum calcium (11.00 +/− 1.29 mg/dl for the Snx10 OC KO mice vs. 11.89 +/− 1.33 mg/dl for the WT, Fig. 7B, left panel) and PTH (0.176 +/− 0.012 ng/ml for the Snx10 OC KO mice vs. 0.142 +/−0.023 ng/ml for the WT, Fig. 7B, right panel). Hematoxylin/eosin and Von Kossa/van Gieson staining of undecalcified femur sections from Snx10 OC KO mice confirmed the presence of mineralized trabeculae within the bone marrow space (Fig. 7C, *top center panel*). However, unlike the Snx10 KD mice, the trabeculae were thoroughly mineralized and not covered by thick layers of unmineralized osteoid (Fig. 7C, *FEMUR center panel*). The growth plate thickness (GpTh, mm) was significantly larger than the WT (0.160 +/− 0.038 mm and 0.092+/− 0.002 mm, P = 0.03, n = 3 per group, S3 Table) and so was the bone volume / tissue volume (BV/TV, %). The osteoid volume per bone volume (OV/BV, %), on the other hand, did not vary significantly between WT and Snx10 OC KO mice confirming that Snx10 OC KO mice are osteopetrotic but not rachitic (S3 Fig.). Histomorphometry performed on sections from lumbar vertebral bodies (Fig. 7C, VERTEBRA panel and S4 Table) and from skull base/floor bones (Fig. 7C, SKULL panel and S5 Table) show similar results.

### Snx10-deficient osteoclasts have impaired extracellular acidification capacity

**Fig 8:**
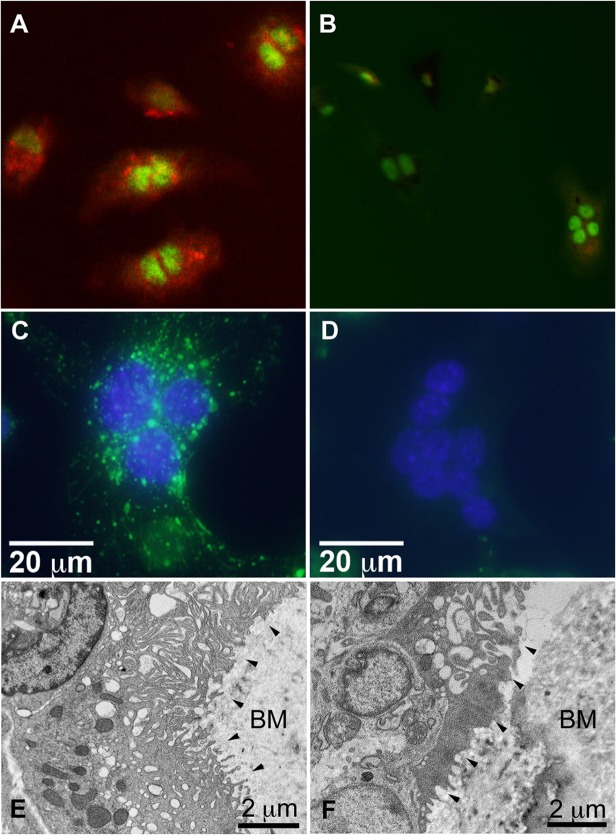
Snx10-deficient osteoclasts are unable to acidify have impaired endosomal pathways, and have defective ruffled borders. Acridine orange indicates acidification by osteoclasts derived from WT splenocytes (A), whereas none is present in Snx10 KD osteoclasts (B)(Nuclei labeled green). (C) WT osteoclasts can internalize fluorescent dextran (green) but Snx10 KD osteoclasts (D) cannot. E) TEM images of bone sections show a well-developed ruffled border in WT osteoclasts (black arrowheads). F) Snx10-deficent osteoclasts form a severely defective ruffled border. BM: bone matrix. (HV = 80.0 kV).

We cultured osteoclast precursors from WT and Snx10 KD mice on dentine slices with M-CSF and RANKL to induce osteoclast differentiation and incubated them with the pH indicator dye, acridine orange. Confocal microscopy revealed the presence of orange fluorescence (i.e. low pH) in WT osteoclasts (Fig. 8A). Orange label was either absent or very faint in Snx10 KD osteoclasts, indicating decreased acidification capacity (Fig. 8B).

### Snx10-deficient osteoclasts have impaired endocytosis and form rudimentary ruffled borders

The endocytic vesicles that contribute to the formation and maintenance of the ruffled border originate in the basolateral membrane of the osteoclast [1]. To study the effect of Snx10 deficiency on endosomal trafficking/vesicle formation and resorption, we performed dextran internalization assays and assessed resorption capacity on HA coated plates. WT cells internalized dextran normally (green punctuate pattern, Fig. 8C). Snx10 KD cells, on the other hand, did not (Fig. 8D). Ultrastructural examination of bone sections (we examined 8 bone sections—4 sections for WT and 4 sections for Snx10 KD—and observed 3 osteoclasts per section) by TEM demonstrated a well-developed ruffled border in WT osteoclasts (Fig. 8E, black arrowheads) whereas a rudimentary ruffled border was observed in Snx10-deficent osteoclasts (Fig. 8F). Thus, Snx10 deficiency prevented endocytosis, severely impaired ruffled border formation, and blocked resorption of HA.

## Discussion

In this study we report that global Snx10-deficient mice (Snx10 KD) die at 3 weeks post-partum and exhibit severe osteopetrosis with a superimposed mineralization defect. Snx10 KD osteoclasts fail to endocytose dextran, form a ruffled border, or acidify the resorption lacuna. Despite these defects, CTX levels are greater in the Snx10 KD deficient mice. This is a seemingly paradoxical result, given that Snx10 deficient-osteoclasts have a severe bone resorption deficiency. However, similar results have been reported for severely osteopetrotic mice with deletions in the osteoclastic anion exchanger 2 (Ae2) [28], and with loss-of-function mutations in the chloride channel 7 (clc-7) [34] and in cathepsin K [30]. This phenomenon may reflect the fact that in osteoclast-rich osteopetrosis, as in these examples, there is a very large increase in bone mass and bone surface area, resulting in a corresponding increase in the total number of osteoclasts. One study of clc-7 KO mice [34] demonstrated that CTX per individual osteoclast was reduced by 50% compared to the WT, but because there also was an increase in the total number of osteoclasts, the total serum CTX was increased.

We also observed a mineralization defect in the Snx10 KD mice, consistent with rickets, suggesting that Snx10 is also required for calcium homeostasis. Pursuing this, we found Snx10 to be expressed in gastric zymogenic cells. Similar to osteoclasts, Snx10 KD zymogenic cells exhibit a defect in secretory vesicle formation/maintenance, leading to hypochlorhydria and hypocalcemia. The Snx10 KD mice thus exhibit a phenotype that is a combination of osteopetrosis (due to impaired osteoclast resorption) and rickets (impaired mineralization due to poor calcium absorption). This is borne out by the correction of the rachitic phenotype when Snx10 is knocked out specifically in osteoclasts, with normalization of stomach pH, circulating calcium, and bone mineralization. Our results are in line with those of Schinke *et al*. [11] who analyzed the combined acid defect phenotype by using mutations in different genes (*Cckbr* for the stomach and *Src* for the osteoclasts). Similarly, the Tcirg1-deficient *oc/oc* mice have an osteopetrorickets phenotype due to impaired acidification in both stomach and bone [11]. These observations unify the complex phenotype seen in Snx10 KD mice and osteopetrorickets patients as being due to the simultaneous inhibition of osteoclast function and gastric acid production. Shinke *et al* [11] reported that 21 patients with diagnosed osteopetrosis had not been genotyped and 10 of these patients had histological evidence of osteopetrorickets that had not been diagnosed. Because calcium supplementation rescued mice from the rachitic phenotype and dramatically extended life-span in Snx10 KD mice, our findings suggest that this may be a critical component of the clinical approach to Snx10-dependent human osteopetrorickets. Because calcium gluconate is taken up at higher pH than calcium carbonte, it is the form of choice.

There is a complex system of cross-regulation that exists between bone and most other organs [35]. Our studies provide further evidence that there are instances of shared molecular machinery for the production of acid in the stomach and in osteoclasts. TreeFam, a database of phylogenetic trees from animal genomes that can be used to infer the evolutionary history of genes, shows that Snx10 appears first in *Osteichthyes*, the bony fishes (http://www.treefam.org/family/TF332117). The acid-secreting stomach also evolved in bony fishes. This raises the intriguing prospect that the osteoclastic and gastric acid producing systems evolved simultaneously during vertebrate evolution, together with emergence of a mineralized skeleton, utilizing some of the same genes.

Snx10 appears to mediate both bone resorption and stomach acidification by regulating vesicular trafficking. Our data and several other reports support a concordance between the secretory trafficking mechanisms in OCs and zymogenic cells. For example, GNPTAB [36–38], the enzyme required for adding mannose-6-phosphate to lysosomal hydrolases, when knocked out in mice, causes defective OC and zymogenic cell trafficking. *Gnptab*-null mice also have low BMD [38,39]. In the stomach, trafficking of digestive enzymes like PGC and acid secretion (and, thus, calcium homeostasis) are linked. Loss of parietal cell acid secretion and/or damage to parietal cells causes loss of mature zymogenic cells [31,40]. It is well-known that the fate and function of parietal and zymogenic cells are profoundly intertwined [41]. Further studies may reveal whether there is Snx10 expression in parietal cells, which may be too low to be seen in the assays used here or if their function depends indirectly on Snx10 expression in zymogenic cells.

Gastric acidification is essential for calcium absorption, and this regulatory pathway may be disrupted in a variety of clinical conditions associated with hypochlorhydria ranging from osteopetrorickets to antacid therapy. Proton-pump inhibitors, which block gastric acid production and increase gastric pH, have recently been associated with hip fracture risk [42,43]. It has been speculated that hypochlorhydria results in impaired calcium absorption thereby increasing fracture risk. Similarly, endosteal bone resorption has been reported following gastric bypass surgery in rodents and humans [44,45]. The marked hyperparathyoirism of the Snx10 KD mice was corrected in the Snx10 OC KO model, consistent with the hypocalcemia in the former which was prevented by normal gastric acdification in the latter. Thus, our findings underscore the relationship between gastric acidification and calcium absorption and its impact on bone health in the general population (for an authoritative review on the topic see [46]), and provide novel insights into the mechanisms governing the regulation of bone accrual via the gastrointestinal tract.

## Materials and Methods

### Targeting vector and mouse models

All animal procedures were approved by IACUC of The Forsyth Institute. We obtained the *Snx10* targeting vector, PG00216_Z_2_C06, from the European Conditional Mouse Mutagenesis Program (EUCOMM). This vector is a “knockout first” gene trap (see S1A Fig.) which inserts a flippase site-flanked Neo selection cassette with an IRES and LacZ reporter into intron 3 and inserts LoxP sites flanking exons 4 and 5. Exons 4 and 5 contain the PX domain required for phospholipid interactions, including all three phospholipid contact amino acid sequences. This allele is designated *Snx10* ^*tm1a(EUCOMM)Hmgu*^. Hereafter in this report, we refer to the resulting targeted allele as *Snx10* ^*Neo-f*^. Mice homozygous for this allele are severely deficient in Snx10 globally (see Results), so we hereafter refer to *Snx10* ^*Neo-f/Neo-f*^ mice as “Snx10 KD,” for Snx10 knockdown.

The gene-trap vector was electroporated into V6.5 ES cells. Neomycin resistant clones were picked, expanded and screened for correct insertion by Long Range Genomic PCR using the following vector-specific primers and gene-specific primers:

5' Integration

Gene Specific Forward (GF3). 5'-GCTTATGGTCGACTCATCGGAGAATC-3'

LacZ Reverse (LAR7), 5'-GGTGTGGGAAAGGGTTCGAAGTTCCTAT-3'

Amplicon size = 5,170 bp

3' Integration

LacZ Forward (LAF), 5'-GAGATGGCGCAACGCAATTAATG-3'

Gene Specific Reverse (GR4),5'-CACAGAAGTAATGTACGCTAATGGCAACG-3'

Amplicon size = 5,680 bp

The resulting clones were injected into host blastocysts to generate mouse chimeras. Two male chimeras were bred with C57BL/6J females. Germ line transmission was confirmed by PCR, using primers flanking the third loxP site (LoxP3 Forward: 5’-ATAACTAACCCAGGCAAACA-3’ and LoxP3 Reverse: 5’-TTGTCAAGTGCGTGTGTCGT-3’; S1A Fig., red arrows). Snx10^*Neo-f/+*^ offspring were bred to homozygosity to generate animals for experiments at the expected Mendelian ratio of approximately 25%. PCR genotyping using the preceding primer pair produced bands of 213 bp for the WT (+/+), 273 bp for the *Snx10* ^*Neo-f/Neo-f*^, and both bands for the heterozygotes (S1B Fig.). For qPCR amplification of cDNA we used the following primers (S1A Fig., black arrows): SNX10 (Forward): 5’-GAACAATCGCCAGCATGTCGAC-3’ and SNX10 (Reverse): 5’-ATGTCC TCGGAGTTCAGATGGC-3’.

Osteoclast-specific Snx10-deficient mice were generated in two steps. First, the Neo cassette was removed by crossing *Snx10* ^*Neo-f/+*^ females with males homozygous for Rosa26-driven FLP recombinase (strain *B6*.*129S4-Gt(ROSA)26Sortm1(FLP1)Dym/RainJ)* [47], resulting in an allele with exons 4 and 5 flanked by loxP sites (*Snx10* ^*f*^). *Snx10* ^*f/f*^ animals were viable and fertile. Then, heterozygous males were crossed with females homozygous for Cre recombinase driven by the cathepsin K promoter (*Ctsk-Cre)* [48]. The resulting mice carry an osteoclast-specific *Snx10* allele with exons 4 and 5 deleted, and therefore a null allele, which we designate *Snx10* ^*OC-*^. *Snx10* ^*OC-/OC-*^ homozygous animals are hereafter referred to as “Snx10 OC KO”, for osteoclast knockout.

### Radiographs and MicroCT

Radiographs were obtained with a Faxitron cabinet radiograph system (Model 43855A, Hewlett Packard, McMinnville, Oregon) with Kodak high-speed holographic film at 40 kV. For micro-CT, mice were sacrificed by CO_2_ asphyxiation. Left femora, tibiae, mandibles and calvariae were then dissected and fixed in 4% paraformaldehyde (PFA, pH = 7.4) at 4°C for 18 hours, followed by 70% ethanol, then stored at 4°C until scanned. Samples were scanned (μCT 40, Scanco Medical) for 3D reconstruction and the following parameters were estimated: BV/TV (bone volume per tissue volume), Tb.N (trabecular number), Tb.Th. (trabecular thickness), Tb.Sp (trabecular spacing), and bone mineral density (BMD). Micro-CT slices, each with a slice thickness reconstructed to 10 μm (100 slices/mm), were obtained delivering a 3-dimensional representation of approximately 3 mm of anatomy. The imaging was done at 55kV, 145μA.

#### Mechanical testing

Previous to the mechanical test, the proximal femur was immersed in acrylic resin until solidification. A loading force was applied to the femoral head using a concave loading cup at a constant speed of 0.155 mm/s until failure. The following parameters were analyzed: maximal load, stiffness, work to failure and maximal displacement. Maximal load reflects the general integrity of the bone structure; stiffness is closely related to the mineralization of the bone; energy to failure is the amount of energy necessary to break the bone; and maximal displacement is inversely related to the brittleness of the bone [25].

### Isolation of spleen cells and osteoclast differentiation

We used splenocytes as the source of osteoclast precursors because long bones in osteopetrotic mice lack marrow cavities. Spleens were resected from 4 week-old mutant and WT mice and mashed through a cell strainer (BD Falcon, Mesh Size: 40μm) To induce osteoclast differentiation, splenocytes were plated in a 24-well plates (1 x 10^6^ cells/well) in alpha-MEM/10% FBS supplemented with M-CSF (25 ng/ml) and RANKL (50 ng/ml)(both from Peprotech). Cells were cultured for 7 days with changes of medium and cytokines every other day.

#### Lentivirus construction

A 605-bp human Snx10 cDNA, containing the entire ORF, was amplified by RT-PCR from RNA from osteoclasts derived from human peripheral blood mononuclear cells (PBMC) using the following primers:

Sense: 5′-GATATGTTTCCGGAACAACAGAAAG-3′

Antisense: 5′-TCAGGATTCCTGCGGAGCTGTATTT-3′

The cDNA was then sub-cloned into the expression vector pLenti6.3/V5-TOPO (Life Technologies Corporation) and was used to generate lentiviral particles using the ViraPower HiPerform Lentiviral TOPO Expression Kit (Invitrogen) according to the manufacturer’s instructions.

### Lentiviral infection

Spleen cells were cultured overnight in alpha-DMEM/10% FBS supplemented with 50ng/ml M-CSF in 24-well plates (1 x 10^6^ cells/well). The next day, the medium was removed and the cells were incubated overnight with lentiviral particle-containing medium in the presence of polybrene (6ug/ml). The following day, the culture medium was replaced with fresh medium containing M-CSF and RANKL. The cells were cultured under osteoclast differentiation conditions for 7 days, with changes of medium and cytokines every other day.

### Pit formation assay

Spleen cells were plated (1 x 10^6^ cells/well) on 24-well Osteo Assay Surface plates (Corning) in alpha-MEM supplemented with 50ng/ml M-CSF. The following day cells were infected with Snx10 lentiviral particles and 24 hours later the medium was replaced with fresh medium containing M-CSF and RANKL. The cells were cultured under osteoclast differentiation conditions for 7 days as above. Cells were then removed by incubation with 10% bleach solution. The wells were washed with PBS, air-dried and photographed under a light microscope. The area of resorption pits was quantified using Image J (National Institutes of Health, Bethesda, MD).

### Dextran internalization

Osteoclast precursors were cultured on bovine dentine slices in alpha-MEM supplemented with M-CSF and RANKL to induce osteoclast differentiation. Differentiated osteoclasts were incubated with 200 μg/ml aldehyde fixable Alexa 488-dextran (Life Technologies, Grand Island, NY, USA) overnight at 37°C. Cultures were fixed in 4% PFA for 15 minutes, counterstained with DAPI (4,6-diamidino-2-phenylindole) to visualize nuclei, and mounted on glass cover slips for fluorescence microscopy. Digital images (obtained using fluorescence microscope FSX100, Olympus) were processed with Image J to obtain an interactive 3D surface plot, where the height of the surface plot reflects image brightness, corresponding to the amount of internalized dextran. Multinucleated cells were counted and each cell was assessed for dextran endocytosis.

### Extracellular acidification

Osteoclasts cultured on dentine slices as above were incubated with 5ug/ml acridine orange (Sigma) for 15 minutes at 37°C, rinsed with PBS and chased for an additional 15 minutes. Images were obtained using a Leica SP5X Laser Scanning Confocal Microscope with a 490-nm excitation filter and a 525-nm emission filter. Digital images were processed with Image J. Multinucleated cells were counted and assessed for the presence of extracellular acidification. Experiments were performed in triplicate and the results were expressed as the proportion of acidification-positive osteoclasts in the total number of osteoclasts (±sd). Differences were analyzed using Student’s *t*-test and considered significant if P<0.05.

### TRAP staining

After culturing cells for 7 days in the presence of RANKL and M-CSF, cultures were washed with PBS, fixed first in 4% PFA for 5 minutes, then briefly in ethanol/acetone, 50%/50%, and air dried for 2 minutes. Cells were then incubated in tartrate resistant acid phosphatase (TRAP) staining solution (Napthol AS-MX phosphate and Fast Red Violet LB Salt) at 37°C until the color developed (10 minutes to 1 hour). The wells were then washed with PBS, air-dried and photographed under a light microscope.

### Histology

Mouse femora were fixed in 4% PFA overnight at 4°C and stored in 50% alcohol at room temperature. For paraffin embedding, the specimens were washed with 5, 10, and 15% glycerol in PBS, each for 15 min and decalcified with 10% EDTA in 0.1M TRIS for 2 weeks. Specimens were then embedded in low melting temperature paraffin and sectioned at 5-μm intervals. Decalcified paraffin sections were used for H&E, IHC and TRAP staining. For plastic embedding, the specimens were dehydrated in a graded series of alcohols then infiltrated and embedded in a methyl and butyl methacrylate resin. Each femur was bisected longitudinally with a precision saw and cross-sections (transverse) were cut at the mid-diaphysis (plane 1) and longitudinal planes in the proximal-to-distal orientation (plane 2). Plane 1 and 2 segments were sectioned using a Leica RM2165 microtome and collected onto coated slides, press-mounted and dried in an oven set at 50°C for several hours. Slides were deplasticized in xylenes, rehydrated in a graded alcohol series into deionized water, stained with Von Kossa-Van Gieson’s stains [11], and covered with a cover slip. Micro-ground sections were prepared from planes 1 and 2 sawed segments, mounted onto plastic slides, then ground and polished to a thickness of <80 microns using the EXAKT CS400 system (EXACT Technologies, Oklahoma City, OK). The micro-ground slides were etched in a solution of 50% acetone + 50% dehydrated ethanol, rinsed in deionized water, then stained with Von Kossa-Van Gieson’s stain, and covered with a cover slip.

Freshly obtained stomach samples were fixed overnight in 4% formalin at 4°C, rinsed in 70% ethanol, arranged in 2% agar in a tissue cassette, and paraffin embedded. 5μm sections were cut, paraffin was removed, and the sections were rehydrated. Antigen retrieval was done for 20 minutes in boiling Trilogy solution (Cell Marque, Rocklin, CA), blocked for 1 hour in 1% bovine serum albumin, 0.3% Triton X-100 in PBS and incubated with primary antibodies overnight. Primary antibodies used were Snx10 (Santa Cruz SC-104657, 1:200), VEGF-B (Santa Cruz SC-1876, 1:250), and GIF (a gift from Dr. David Alpers, Washington University School of Medicine, 1:2000). Secondary antibodies, GSII lectin and Hoechst 33358 labeling were as described [32]

### Morphometric methodology

Using morphometric software (i.e., Olympus MicroSuite Biological Suite or cellSens), five high magnification images of methylmethacrylate sections were acquired from the sub-articular region (i.e., immediately subjacent to the growth plate) of the proximal femur (40x), the bones of the skull base/floor (i.e., basisphenoid) (40x) and lumbar vertebral bodies (20x). Calibrated images of the femora, calvariae, and vertebrae were then morphometrically assessed to determine: osteoid volume/bone volume (OV/BV, %) and bone volume/tissue volume (BV/TV, %), and, in addition, the growth plate thickness (GpTh, mm) of distal femur, all as described [49]. Bone and osteoid volume were calculated based on the summation of all five sampling fields. Ratios (i.e., OV/BV and BV/TV) are presented as the average of all five fields. Since our morphometry was done in 2D we measured areas and then calculated volumes (i.e., by multiplying perimeter area values 4/π to getsurface/volume). Data were consolidated and presented as mean ± sd

### Transmission electron microscopy

Mice were perfused with 4% (w/v) PFA and 1% (v/v) glutaraldehyde in 0.1 M phosphate buffer (pH 7.4). Femora were isolated, defleshed and immediately fixed in 4% (w/v) PFA and 1% (v/v) glutaraldehyde in 0.1 M phosphate buffer (pH 7.4) at 4°C for 1 h prior to cutting the femur into approximately 1 mm slices, which continued to be fixed for additional 24 h and then rinsed in 0.1M sodium phosphate buffer. Bone slices were decalcified in 10% (w/v) EDTA in PBS for 3 days, post fixed in 1% (v/v) OsO_4_ and embedded in Epon-812 resin (Tousimis, Rockville, MD). Ultrathin sections (n = 4 per group) were then made and stained with uranyl acetate and lead citrate, and examined with a FEI Tecnai G2 Spirit Transmission Electron Microscope.

### Measurement of gastric pH

Gastric pH was measured as described [50]. Briefly, mice were fasted for 2 hours prior to the test and anesthetized. The stomach was then exposed after abdominal midline incision, followed by ligation of the pylorus and esophagus. Saline solution was then injected into the stomach and the fluids collected. The pH of the collected fluid was measured using a Star A321 pH Meter (Thermo Scientific Orion) equipped with a PerpHecT ROSS pH electrode with micro tip (Thermo Scientific Orion).

### Determination of serum calcium, PTH and 1,25- dihydroxyvitamin D_3_

For analysis of calcium homeostasis, blood was obtained by cardiac puncture and collected in heparinized Eppendorf tubes. Serum was isolated by centrifugation at 3000 g for 10 min and stored at −80°C. Serum calcium was measured by the Clinical & Epidemiologic Research Laboratory at Children’s Hospital (Boston, MA), a nationally certified laboratory, using a colorimetric assay on a Hitachi 917 analyzer using Roche reagents (Roche Diagnostics, Indianapolis, IN). The lowest detection limit of this assay is 0.2 mg/dL and the day-to-day imprecision values at concentrations of 8.38, 9.12 and 13.13 mg/dL are 1.5%, 1.6%, and 0.8%, respectively. PTH was determined using the Mouse PTH 1–84 ELISA Kit (Cat.# 60-2305, Immutopics, Inc., San Clemente, CA). *1*,*25- dihydroxyvitamin D* was determined using the Mouse Vitamin D, VD ELISA Kit (CSB-E07912m, Cusabio Life Science).

### Determination of markers of bone formation and resorption

Quantification of fragments of type I collagen in serum was done using the RatLap EIA kit from IDS (Immunodiagnostic Systems, AC-06F1) following the manufacturer's instructions. Serum osteocalcin was measured using the Mouse Osteocalcin ELISA Kit from Immutopics (60-1305), following the manufacturer's instructions.

## Supporting Information

S1 Fig Generation of Snx10 KD mice. A) Vector PG00216_Z_2_C06. Insertion of this vector into the Snx10 locus by homologous recombination produces an allele of the type: “Knockout-First—Reporter Tagged Insertion” (Promoter Driven Cassette). B) PCR genotyping of *Snx10* ^*+/+*^ (WT), *Snx10* ^*Neo-f/+*^, and *Snx10* ^*Neo-f/Neo-f*^ (Snx10 KD) mice. C) Snx10 KD mice are Snx10-deficient. Snx10 expression in bone is reduced by ∼86% in Snx10 KD mice (relative expression Snx10 KD = 0.14 vs. WT = 1.03, n = 4 per group, P<0.05).
(TIF)

S2 Fig Snx10 deficiency inhibits osteoclast formation and activity *in vivo* and *in vitro*. A) TRAP staining (red) of longitudinal tibia sections from Snx10 KD mice show that Snx10 KD mice have osteoclasts (magnification 10X). Splenocytes from WT (B) or Snx10 KD (C) mice can generate TRAP+ osteoclasts after *ex vivo* stimulation. D) Osteoclasts derived from WT, but not from Snx10 KD splenocytes, can resorb hydroxyapatite (top and middle panel). Infection of Snx10 KD splenocytes with a virus expressing Snx10 (Snx10 KD + Snx10), corrects the defective resorption phenotype (* P < 0.05)
(TIF)

S3 Fig Snx10 KD and Atp6i KO mice show a combined phenotype: osteopetrorickets. A) Radiographs (top and bottom panels) show that Snx10 KD and Atp6i KO mice have bones with a higher radio-density and lack cortical bone. The metaphyseal fraying and cupping seen in the femur and tibia are features of rickets (bottom panels). B) Micro-CT surface images of the femur show cortical bone deficiency, which produces a “moth-eaten” appearance and also confirms hypo-mineralization of condyles in both mutants. The white line in the longitudinal mid-plane sections (B, top) indicates the region where the transverse micro-CT images were taken (B, bottom).
(TIF)

S4 Fig Snx10 KD mice displayed reduced zymogenic cell granular size and abundance. A) IF images from stomach sections from WT and Snx10 KD mice stained for GIF (a zymogenic cell marker, ZC), Snx10, GSII (a marker for the mucous neck cells interspersed with the parietal cells in the neck of the gastric unit) shows Snx10-specific staining in WT zymogenic cells. B) H&E confirms the small size and high nuclear:cytoplasmic ratio of parietal cells in KO gastric units (also noted in Fig. 5D). The ZCs, lacking their abundant apical granules, are also smaller, and there are increased parietal cells in the base (ZC) zone (black arrowheads). This zymogenic defect phenotype resembles the *Mist1* KO mouse.
(TIF)

S1 Table Mechanical properties of diaphyseal tibiae from WT and Snx10 KD mice.
(DOCX)

S2 Table FEMUR histomorphometry: WT and Snx10 KD (3.5 week-old mice).
(DOCX)

S3 Table FEMUR histomorphometry: WT and Snx10 OC KO (6 week-old mice).
(DOCX)

S4 Table VERTEBRA histomorphometry: WT, Snx10 OC KO and Snx10 KD (6 week-old mice).
(DOCX)

S5 Table SKULL histomorphometry: WT, Snx10 OC KO and Snx10 KD (6 week-old mice).
(DOCX)

S6 Table FEMUR histomorphometry: Calcium supplementation of Snx10 KD mice (111 day old mice, Femur).
(DOCX)
