# Supplementary figures and images for: Osteopetrorickets due to Snx10 Deficiency in Mice Results from Both Failed Osteoclast Activity and Loss of Gastric Acid-Dependent Calcium Absorption
Source: PLoS Genet. 2015 Mar 26;11(3):e1005057. doi: 10.1371/journal.pgen.1005057 (PMC4374855; doi:10.1371/journal.pgen.1005057)

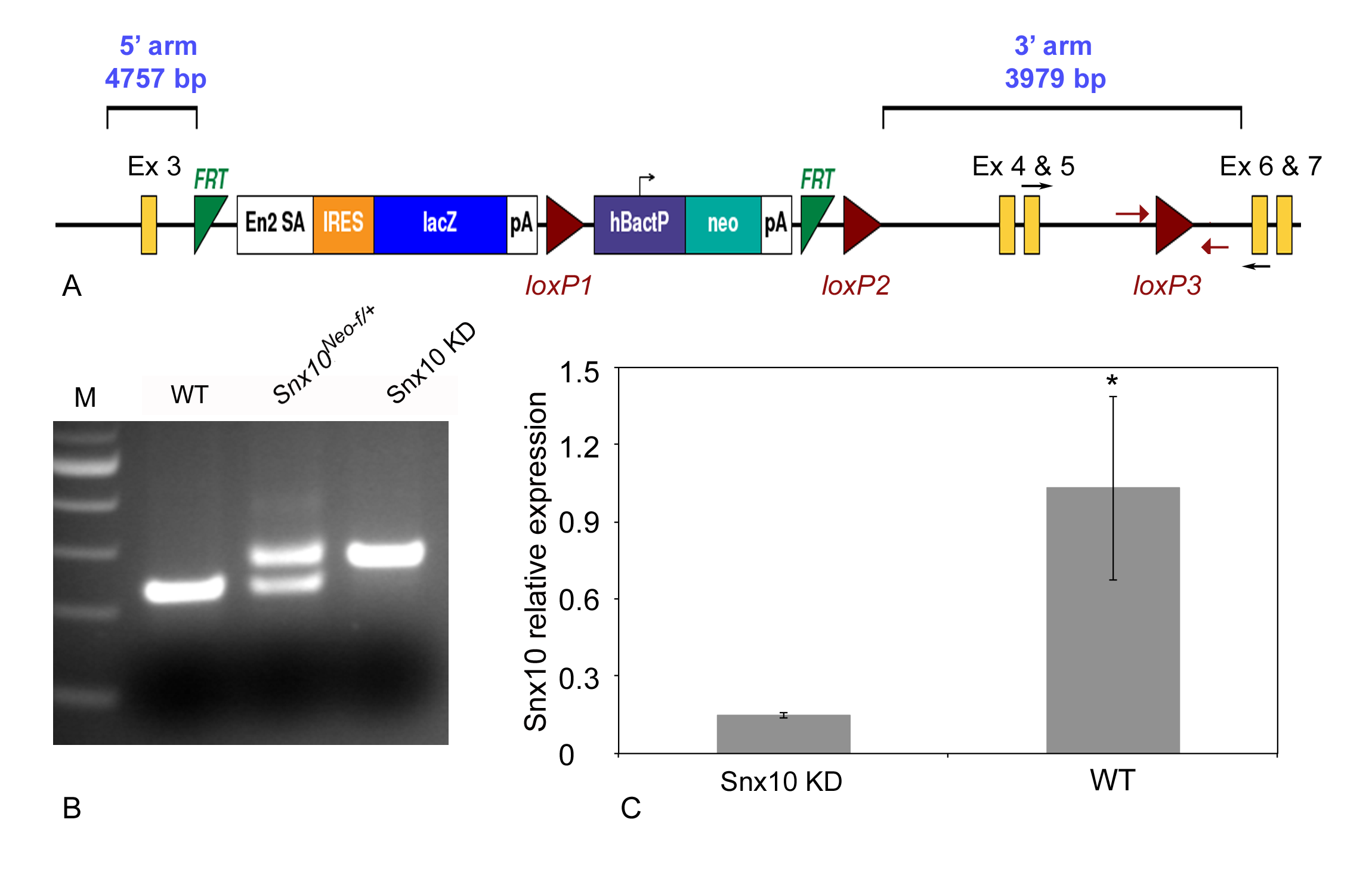

Supplement: S1 Fig — Generation of Snx10 KD mice. A) Vector PG00216_Z_2_C06. Insertion of this vector into the Snx10 locus by homologous recombination produces an allele of the type: “Knockout-First—Reporter Tagged Insertion” (Promoter Driven Cassette). B) PCR genotyping of Snx10 +/+ (WT), Snx10 Neo-f/+, and Snx10 Neo-f/Neo-f (Snx10 KD) mice. C) Snx10 KD mice are Snx10-deficient. Snx10 expression in bone is reduced by ∼86% in Snx10 KD mice (relative expression Snx10 KD = 0.14 vs. WT = 1.03, n = 4 per group, P<0.05). (TIF) [file pgen.1005057.s001.tif]

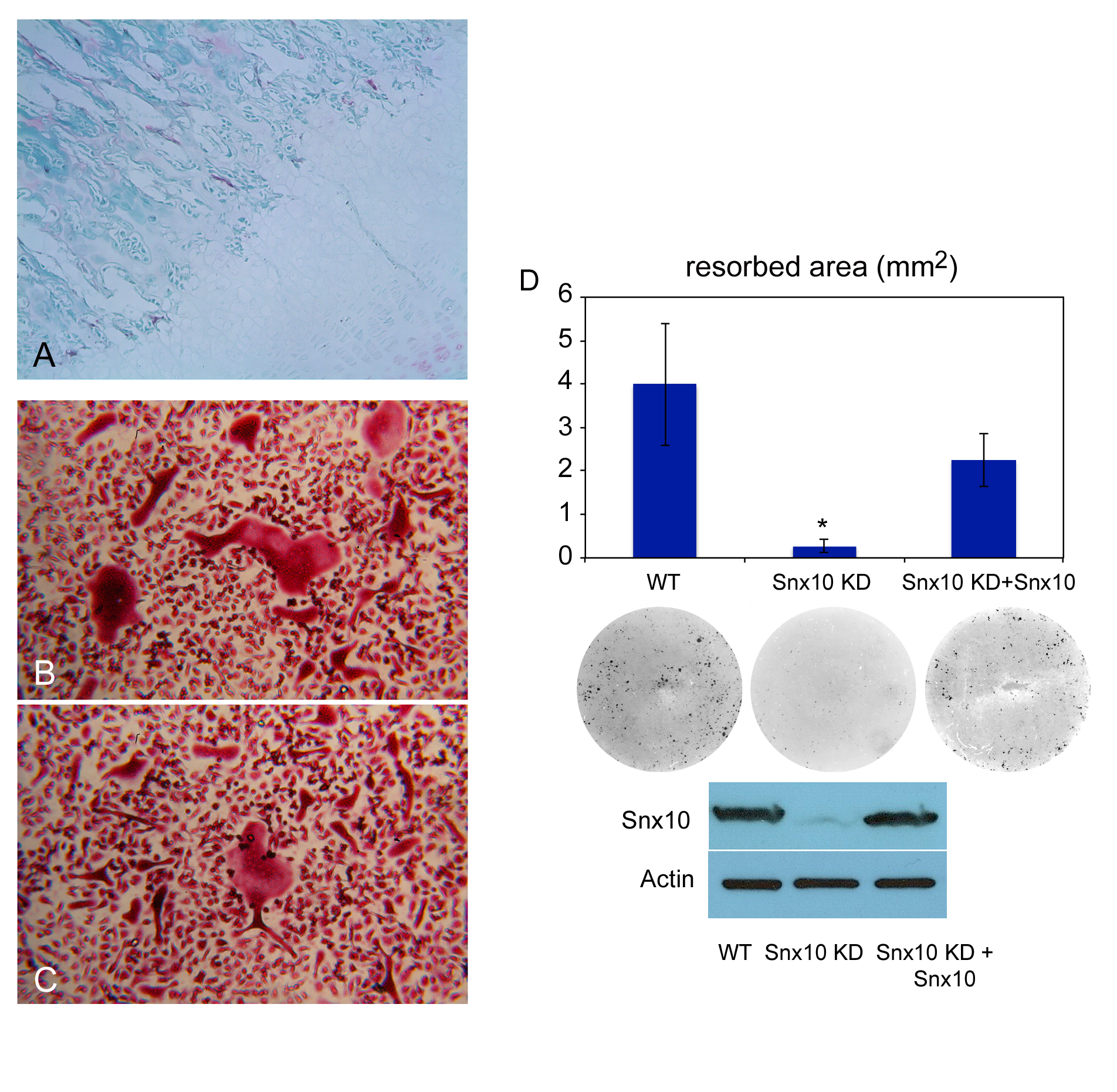

Supplement: S2 Fig — Snx10 deficiency inhibits osteoclast formation and activity in vivo and in vitro. A) TRAP staining (red) of longitudinal tibia sections from Snx10 KD mice show that Snx10 KD mice have osteoclasts (magnification 10X). Splenocytes from WT (B) or Snx10 KD (C) mice can generate TRAP+ osteoclasts after ex vivo stimulation. D) Osteoclasts derived from WT, but not from Snx10 KD splenocytes, can resorb hydroxyapatite (top and middle panel). Infection of Snx10 KD splenocytes with a virus expressing Snx10 (Snx10 KD + Snx10), corrects the defective resorption phenotype (* P < 0.05) (TIF) [file pgen.1005057.s002.tif]

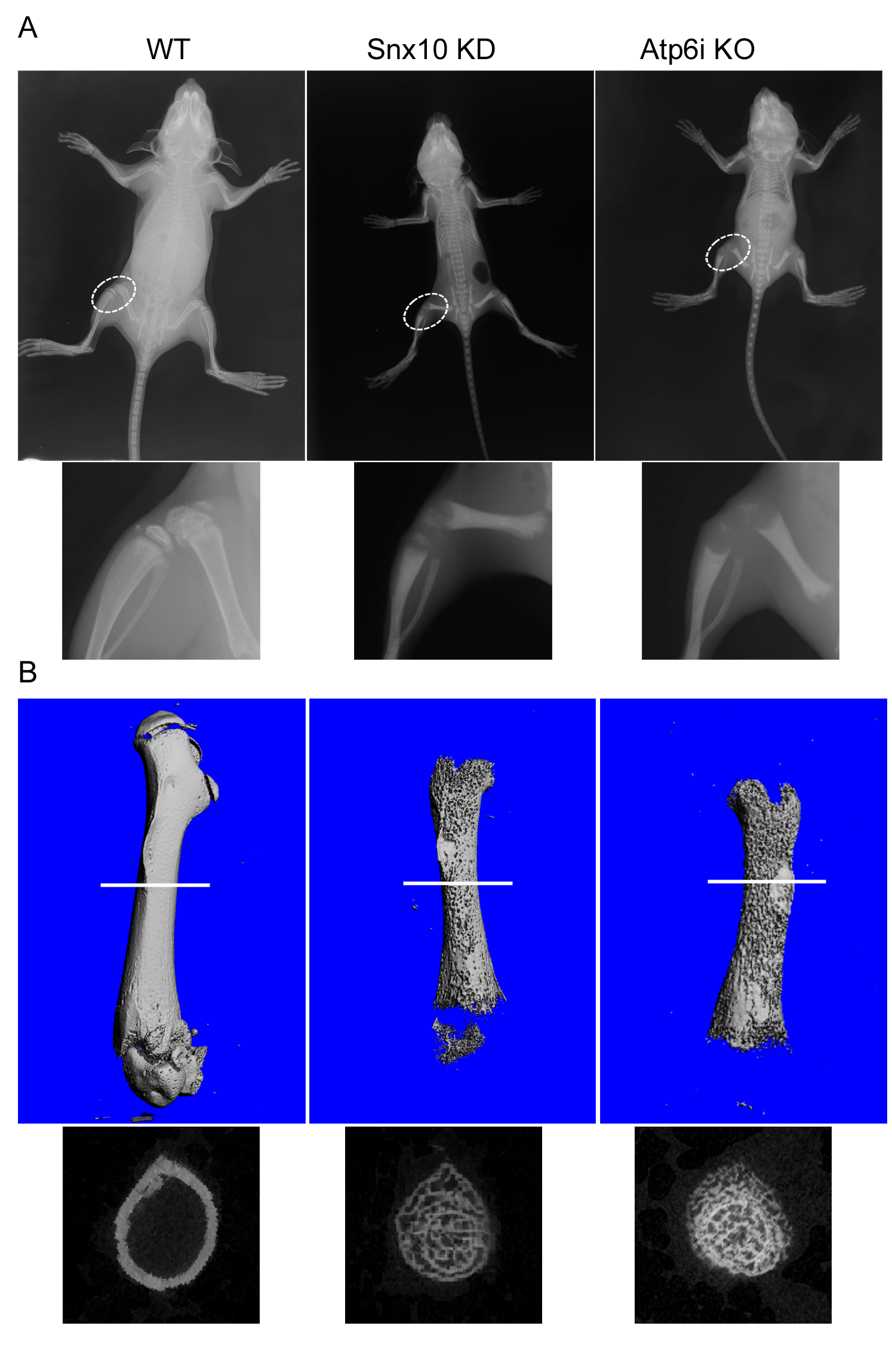

Supplement: S3 Fig — Snx10 KD and Atp6i KO mice show a combined phenotype: osteopetrorickets. A) Radiographs (top and bottom panels) show that Snx10 KD and Atp6i KO mice have bones with a higher radio-density and lack cortical bone. The metaphyseal fraying and cupping seen in the femur and tibia are features of rickets (bottom panels). B) Micro-CT surface images of the femur show cortical bone deficiency, which produces a “moth-eaten” appearance and also confirms hypo-mineralization of condyles in both mutants. The white line in the longitudinal mid-plane sections (B, top) indicates the region where the transverse micro-CT images were taken (B, bottom). (TIF) [file pgen.1005057.s003.tif]

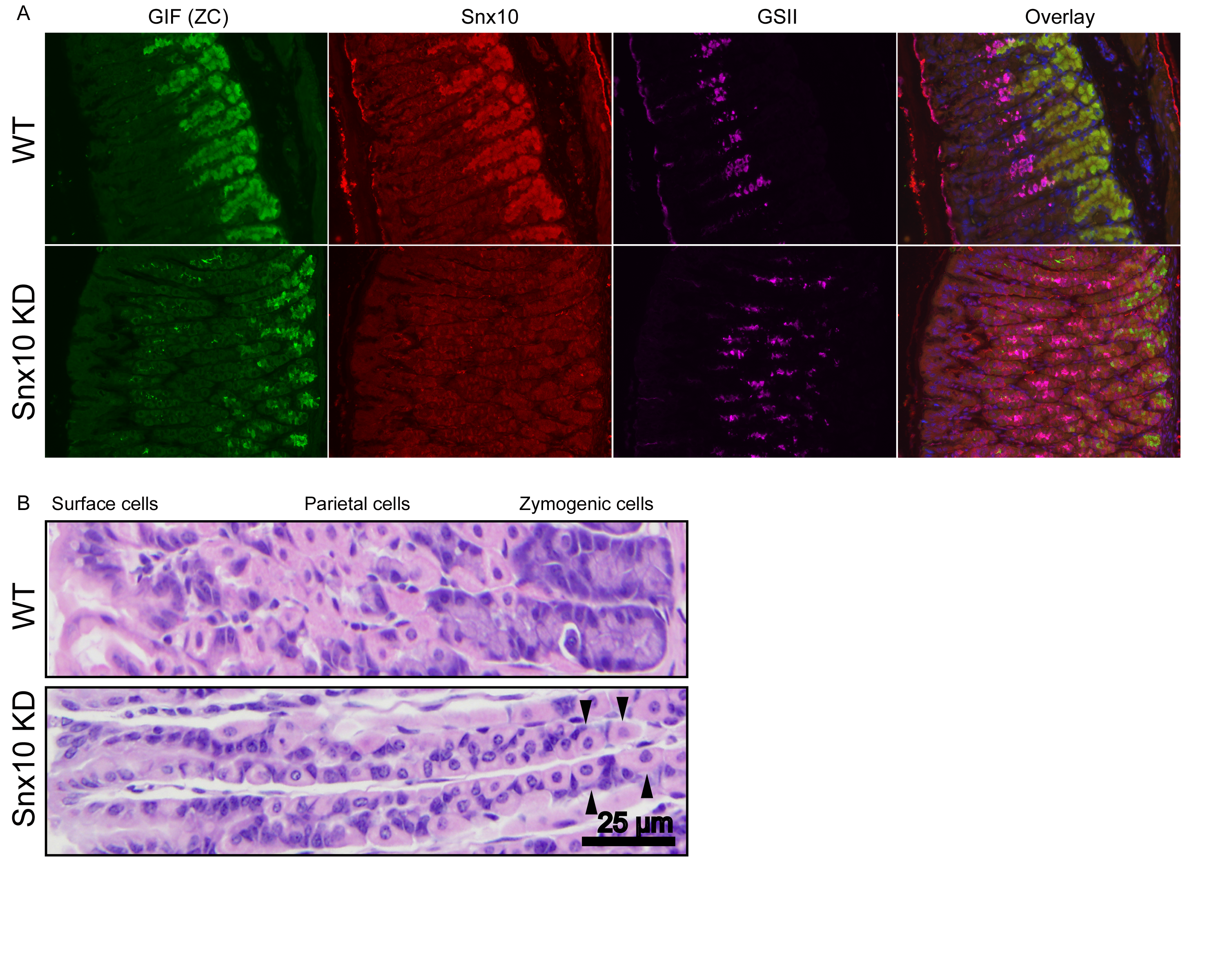

Supplement: S4 Fig — Snx10 KD mice displayed reduced zymogenic cell granular size and abundance. A) IF images from stomach sections from WT and Snx10 KD mice stained for GIF (a zymogenic cell marker, ZC), Snx10, GSII (a marker for the mucous neck cells interspersed with the parietal cells in the neck of the gastric unit) shows Snx10-specific staining in WT zymogenic cells. B) H&E confirms the small size and high nuclear:cytoplasmic ratio of parietal cells in KO gastric units (also noted in Fig. 5D). The ZCs, lacking their abundant apical granules, are also smaller, and there are increased parietal cells in the base (ZC) zone (black arrowheads). This zymogenic defect phenotype resembles the Mist1 KO mouse. (TIF) [file pgen.1005057.s004.tif]
