# Supplementary material for: Osteopetrorickets due to Snx10 Deficiency in Mice Results from Both Failed Osteoclast Activity and Loss of Gastric Acid-Dependent Calcium Absorption
Source: PLoS Genet. 2015 Mar 26;11(3):e1005057. doi: 10.1371/journal.pgen.1005057 (PMC4374855; doi:10.1371/journal.pgen.1005057)
Supplement: S1 Table — Mechanical properties of diaphyseal tibiae from WT and Snx10 KD mice. (DOCX) [file pgen.1005057.s005.docx]

S1 Table. Mechanical properties of diaphyseal tibiae from WT and Snx10 KD mice

|  | WT | *Snx10 KD* | *P* value |
| --- | --- | --- | --- |
| Maximal Load (N) | 10.27 ± 0.97 | 2.78 ± 1.66 | 0.0025 |
| Stiffness (N/mm) | 46.68 ± 1.16 | 9.71 ± 7.33 | 0.001 |
| Energy to failure (Nmm) | 0.34 ± 0.08 | 0.15 ± 0.06 | 0.027 |
| Maximal displacement (mm) | 0.38 ± 0.07 | 0.58 ± 0.10 | 0.05 |

n=6
