# Supplementary material for: Osteopetrorickets due to Snx10 Deficiency in Mice Results from Both Failed Osteoclast Activity and Loss of Gastric Acid-Dependent Calcium Absorption
Source: PLoS Genet. 2015 Mar 26;11(3):e1005057. doi: 10.1371/journal.pgen.1005057 (PMC4374855; doi:10.1371/journal.pgen.1005057)
Supplement: S2 Table — FEMUR histomorphometry: WT and Snx10 KD (3.5 week-old mice). (DOCX) [file pgen.1005057.s006.docx]

S2 Table. FEMUR Histomorphometry: WT and Snx10 KD (3.5 week-old mice)

|  | *WT* | *Snx10 KD* |
| --- | --- | --- |
| Growth Plate Thickness  (GpTh, mm) | 0.175 | 0.286 |
| sd | 0.010 | 0.062 |
| P = 0.04, n=3 per group |  |  |
|  | *WT* | *Snx10 KD* |
| Osteoid volume per Bone volume (OV/BV, %) | 2.88 | 26.97 |
| sd | 1.63 | 8.19 |

P = 0.01, n=3 per group

|  | *WT* | *Snx10 KD* |
| --- | --- | --- |
| Bone volume / Tissue volume (BV/TV, %) | 20.69 | 28.11 |
| sd | 2.15 | 0.98 |

P = 0.02, n=3 per group
