# Supplementary material for: Osteopetrorickets due to Snx10 Deficiency in Mice Results from Both Failed Osteoclast Activity and Loss of Gastric Acid-Dependent Calcium Absorption
Source: PLoS Genet. 2015 Mar 26;11(3):e1005057. doi: 10.1371/journal.pgen.1005057 (PMC4374855; doi:10.1371/journal.pgen.1005057)
Supplement: S3 Table — FEMUR histomorphometry: WT and Snx10 OC KO (6 week-old mice). (DOCX) [file pgen.1005057.s007.docx]

S3 Table. FEMUR Histomorphometry: WT and Snx10 OC KO (6 week-old mice)

|  | *WT* | *Snx10 OC KO* |
| --- | --- | --- |
| Growth Plate Thickness  (GpTh, mm) | 0.092 | 0.1603 |
| sd | 0.002 | 0.0388 |
| P = 0.03, n=3 per group |  |  |
|  | *WT* | *Snx10 OC KO* |
| Osteoid volume per Bone volume (OV/BV, %) | 4.78 | 2.48 |
| sd | 1.34 | 1.33 |

P = 0.1, n=3 per group

|  | *WT* | *Snx10 OC KO* |
| --- | --- | --- |
| Bone volume / Tissue volume (BV/TV, %) | 24.67 | 51.41 |
| sd | 5.82 | 4.27 |

P = 0.003, n=3 per group
