# Supplementary material for: Osteopetrorickets due to Snx10 Deficiency in Mice Results from Both Failed Osteoclast Activity and Loss of Gastric Acid-Dependent Calcium Absorption
Source: PLoS Genet. 2015 Mar 26;11(3):e1005057. doi: 10.1371/journal.pgen.1005057 (PMC4374855; doi:10.1371/journal.pgen.1005057)
Supplement: S4 Table — VERTEBRA histomorphometry: WT, Snx10 OC KO and Snx10 KD (6 week-old mice). (DOCX) [file pgen.1005057.s008.docx]

S4 Table. VERTEBRA Histomorphometry: WT, Snx10 OC KO and Snx10 KD (6 week-old mice)

|  | *WT* | *Snx10 OC KO* | *Snx10 KD* |
| --- | --- | --- | --- |
| Osteoid volume per  Bone volume (OV/BV,%) | 0.48 | 0.89 | 0.98 |
| sd | 0.68 | 1.40 | 0.85 |

P (WT vs. Snx10 OC KO) = 0.73, P (WT vs. Snx10 KD) = 0.54,

n=3 per group

|  | *WT* | *Snx10 OC KO* | *Snx10 KD* |
| --- | --- | --- | --- |
| Bone volume / Tissue volume (BV/TV, %) | 13.95 | 39.50 | 25.98 |
| sd | 1.28 | 5.11 | 5.79 |

P (WT vs. Snx10 OC KO) = 0.007, P (WT vs. Snx10 KD) = 0.05,

n=3 per group
