# Supplementary material for: Osteopetrorickets due to Snx10 Deficiency in Mice Results from Both Failed Osteoclast Activity and Loss of Gastric Acid-Dependent Calcium Absorption
Source: PLoS Genet. 2015 Mar 26;11(3):e1005057. doi: 10.1371/journal.pgen.1005057 (PMC4374855; doi:10.1371/journal.pgen.1005057)
Supplement: S5 Table — SKULL histomorphometry: WT, Snx10 OC KO and Snx10 KD (6 week-old mice). (DOCX) [file pgen.1005057.s009.docx]

S5 Table. SKULL Histomorphometry: WT, Snx10 OC KO and Snx10 KD (6 week-old mice)

|  | *WT* | *Snx10 OC KO* | *Snx10 KD* |
| --- | --- | --- | --- |
| Osteoid volume per  Bone volume (OV/BV,%) | 0.40 | 2.94 | 4.14 |
| sd | 0.41 | 2.54 | 0.87 |

P (WT vs. Snx10 OC KO) = 0.16, P (WT vs. Snx10 KD) = 0.002,

n=3 per group

|  | *WT* | *Snx10 OC KO* | *Snx10 KD* |
| --- | --- | --- | --- |
| Bone volume / Tissue volume (BV/TV, %) | 13.48 | 36.16 | 33.31 |
| sd | 0.37 | 4.92 | 6.09 |

P (WT vs. Snx10 OC KO) = 0.001, P (WT vs. Snx10 KD) = 0.004,

n=3 per group
