# Supplementary material for: Osteopetrorickets due to Snx10 Deficiency in Mice Results from Both Failed Osteoclast Activity and Loss of Gastric Acid-Dependent Calcium Absorption
Source: PLoS Genet. 2015 Mar 26;11(3):e1005057. doi: 10.1371/journal.pgen.1005057 (PMC4374855; doi:10.1371/journal.pgen.1005057)
Supplement: S6 Table — FEMUR histomorphometry: Calcium supplementation of Snx10 KD mice (111 day old mice, Femur). (DOCX) [file pgen.1005057.s010.docx]

S6 Table. FEMUR Histomorphometry: Calcium supplementation of Snx10 KD mice (111 day old mice, Femur)

|  | *WT* | *Snx10 KD + Ca* |
| --- | --- | --- |
| Growth Plate Thickness  (GpTh, mm) | 0.080 | 0.079 |
| sd | 0.043 | 0.018 |
| P = 0.99, n=3 per group |  |  |
|  | *WT* | *Snx10 KD + Ca* |
| Osteoid volume per Bone volume (OV/BV, %) | 3.28 | 7.72 |
| sd | 1.38 | 10.72 |

P = 0.61, n=3 per group

|  | *WT* | *Snx10 KD + Ca* |
| --- | --- | --- |
| Bone volume / Tissue volume (BV/TV, %) | 28.35 | 64.21 |
| sd | 8.68 | 6.31 |

P = 0.04, n=3 per group
